# Effect of Pretreatments on the Chemical, Bioactive and Physicochemical Properties of *Cinnamomum camphora* Seed Kernel Extracts

**DOI:** 10.3390/foods13132064

**Published:** 2024-06-28

**Authors:** Pengbo Wang, Zhixin Wang, Manqi Zhang, Xianghui Yan, Jiaheng Xia, Junxin Zhao, Yujing Yang, Xiansi Gao, Qifang Wu, Deming Gong, Ping Yu, Zheling Zeng

**Affiliations:** 1 State Key Laboratory of Food Science and Resources, Nanchang University, Nanchang 330047, China; 5804117050@email.ncu.edu.cn (P.W.); xia_jheng@163.com (J.X.); junxinzhao@hotmail.com (J.Z.); 17698319443@163.com (Y.Y.); 352800230033@email.ncu.edu.cn (X.G.); kawhi20@163.com (Q.W.); dgong01@gmail.com (D.G.); 2 Jiangxi Province Key Laboratory of Edible and Medicinal Resources Exploitation, Nanchang University, Nanchang 330031, China; 3 School of Chemistry and Chemical Engineering, Nanchang University, Nanchang 330031, China; zhixinwang2003@hotmail.com (Z.W.); 5812120118@email.ncu.edu.cn (M.Z.); xianghui_y@163.com (X.Y.); 4 School of Food Science and Technology, Nanchang University, Nanchang 330031, China; 5 New Zealand Institute of Natural Medicine Research, 8 Ha Crescent, Auckland 2104, New Zealand

**Keywords:** antioxidant, by-product, *Cinnamomum camphora* seed kernel, lipid emulsion, polar bioactive substances, pretreatment method

## Abstract

*Cinnamomum camphora* seed kernels (CCSKs) are rich in phytochemicals, especially plant extracts. Phytochemicals play a vital role in therapy due to their strong antioxidant and anti-inflammatory activities. Extracts from CCSK can be obtained through multiple steps, including pretreatment, extraction and purification, and the purpose of pretreatment is to separate the oil from other substances in CCSKs. However, *C. camphora* seed kernel extracts (CKEs) were usually considered as by-products and discarded, and their potential bioactive values were underestimated. Additionally, little has been known about the effect of pretreatment on CKE. This study aimed to investigate the effects of pretreatment methods (including the solvent extraction method, cold pressing method, aqueous extraction method and sub-critical fluid extraction method) on the extraction yields, phytochemical profiles, volatile compounds and antioxidant capacities of different CKE samples. The results showed that the CKE samples were rich in phenolic compounds (15.28–20.29%) and alkaloids (24.44–27.41%). The extraction yield, bioactive substances content and in vitro antioxidant capacity of CKE pretreated by the sub-critical fluid extraction method (CKE-SCFE) were better than CKEs obtained by other methods. CKE pretreated by the solvent extraction method (CKE-SE) showed the best lipid emulsion protective capacity. Moreover, the volatile substances composition of the CKE samples was greatly influenced by the pretreatment method. The results provided a fundamental basis for evaluating the quality and nutritional value of CKE and increasing the economic value of by-products derived from CCSK.

## 1. Introduction

Plant extracts are often valued due to their high bioavailability and low toxicity, and they have been widely used in the treatment of diseases such as arthritis and hyperlipidemia [1]. CKE exerts varieties of bioactivities such as antibacterial activity [2], anti-tumor activity [3] and antioxidant capacity [4].

*Cinnamomum camphora* L. is widely distributed in southern China, South Korea, Japan and other subtropical Asian countries [5]. It was reported that the annual yield of *C. camphora* seeds exceed 1 million tons in China [6]. The main components of *C. camphora* seed kernels (CCSKs) are lipids (56.00–59.34%), protein (18.15–19.34%) and carbohydrate (10.91–17.90%) [7,8]. Additionally, there are abundant bioactive components in CCSKs. The phytochemicals in CKE include phenolic acids, flavonoids, coumarins, saponins, alkaloids and organic acids [7,9]. However, these by-products in CCSK were often discarded, and their potential bioactive values were underestimated.

CKE from CCSKs is obtained through multiple steps, including pretreatment, extraction and purification, and the purpose of pretreatment is to separate CCSKO from other substances in CCSKs [7,10]. There are many pretreatment methods for CCSKs, including the conventional solvent extraction (SE) method and pressure extraction (PE) method, as well as the burgeoning aqueous extraction (AE) method and sub-critical fluid extraction (SCFE) method [8]. The SE method is extensively applied in oil extraction because of its high oil recovery, although organic solvent may exert a negative impact on both humans and the environment [11]. PE methods include cold pressure (CP) and hot pressure (HP), which are known as safe and environmentally friendly ways to produce oil, while the main problem is their low oil yield [12]. Based on the insolubility of oil, the AE method takes water as the extraction medium, which is apparently safe, eco-friendly and economical [13]. The SCFE method is widely accepted for its low temperature requirements, as well as its use of non-toxic and volatile solvents (butane or propane) [14,15]. However, the bioactive substances from oilseeds are often unstable, and their yield, chemical composition and physicochemical properties may change due to the influence of technological parameters (such as operative temperature and the specific surface area of the obtained material) in different pretreatment methods, which may limit their application in the food and pharmaceutical industries [16]. Additionally, little has been known about the effect of pretreatment on CKE.

This study thus aimed to investigate the effect of pretreatment on CKE. The qualities of four CKE samples prepared by different pretreatment methods, including extraction efficiency, chemical composition, volatile compounds, physicochemical properties and bioactivities, were compared. The results provide a fundamental basis for evaluating the quality and nutritional value of CKE and increasing the economic value of by-products derived from CCSKs.

## 2. Materials and Methods

### 2.1. Chemicals and Materials

The *C. camphora* seeds were collected in the campus of Nanchang University, Jiangxi Province, China, in December 2023. The ABTS, TPTZ, Trolox, CH, BHT, TEP and TBA were purchased from Shanghai Aladdin Biochemical Technology Co., Ltd. (Shanghai, China). (All of the abovementioned chemicals and reagents used were of analytical grade). AB-8 macroporous resin, folin-phenol, gallic acid (HPLC grade), rutin (HPLC grade) and ginsenoside-Rb1 (HPLC grade) were purchased from Beijing Solarbio Science & Technology Co., Ltd. (Beijing, China). The formic acid (HPLC grade), acetonitrile (HPLC grade) and methyl alcohol (HPLC grade) were from Tedia Company, Inc. (Fairfield, OH, USA). The DPPH was from Tokyo Chemical Industry Co., Ltd. (Tokyo, Japan). All other chemicals and reagents used were of analytical grade.

### 2.2. Sample Pretreatment

According to Wang et al. [17], CCSK was obtained through removing the pericarp of *C. camphora* seeds, grading based on diameters with vibration screening machine, breaking the testae of the seed cores with three-roll pressing shellers, and removing the broken testae based on density and color difference with the kernel and testa separator. The diameter of the CCSKs was 3–4 mm, and the color of the CCSKs ranged from faint yellow to light brown. The obtained CCSKs were stored at 4 °C until further use.

The four aliquots of CCSK (200 g each in triplicate) were pretreated by four different oil extraction methods (SE, AE, CP and SCFE), respectively.

SE: The solvent extraction method was performed according to Yan et al. [10], with slight modification. Briefly, the CCSK was crushed using a pulverizer and defatted with n-hexane at the ratio of 1:3 (*m*/*v*) at room temperature. The mixture was continuously stirred for 4 h and repeated three times, then separated by vacuum filtration. The obtained residue was air-dried at room temperature. The defatted dried flour was sieved through a 40-mesh screen and stored at 4 °C until further use.

AE: According to Zhu et al. [8], the CCSK was mixed with water at the ratio of 1:4 (*m*/*v*) in a colloid grinder (XJT-L50A, Guangzhou, China). The mixture was centrifuged at 4000 rpm for 15 min, then the aqueous fraction was collected and stored at 4 °C until further use.

CP: The cold pressing method was carried out following the method of Miao et al. [18]. The CCSK was ground and manually pressed by a screw press (SUNZM23, Shenzhen, China) at room temperature; the pressed cake was collected and stored at 4 °C until further use.

SCFE: Following the method of Shi et al. [19], the CCSK was crushed into fine powder and put into a stainless steel extraction vessel (Sub-critical Extraction Biotechnology Co., Ltd., Henan, China) with butane in a ratio of 1:5 (*m*/*v*). The extraction temperature, pressure and time were set at 40 °C, 0.5 MPa and 2 h. The defatted powder was collected and stored at 4 °C until further use.

Each sample was batch-triplicated according to the abovementioned operating conditions.

### 2.3. Preparation and Purification of Ethanol Extracts

The defatted CCSKs obtained by the SE, CP and SCFE methods were dispersed in 80% (*v*/*v*) ethanol at a ratio of 1:20 (*w*/*v*) and fully stirred at room temperature for 2 h, repeated three times. The mixture was filtered through vacuum filtration. The filtrates were combined and concentrated in a vacuum rotary evaporator at 45 °C. After being lyophilized at −80 °C for 48 h, the CCSK ethanol extract was stored at −20 °C for further use.

Following the procedure previously described by Cheng et al. [20], four samples were dissolved in Milli-Q water, and vacuum filtration was applied to acquire the filtrate for later use. The activated AB-8 macroporous resin was rinsed with water and continuously dispersed by approximately 150 mL of water. The four defatted CCSK samples were absorbed by the loaded AB-8 macroporous resin and then eluted by 70% (*v*/*v*) ethanol. Finally, the eluents were concentrated in a vacuum rotary evaporator at 45 °C, followed by lyophilization and storage at −20 °C.

### 2.4. Yield Calculation

The CKEs prepared by four different pretreatment methods were weighed using an analytical balance (BSA223S, Sartorius Corporate, Göttingen, Germany). The yields of defatted powder and CKEs were calculated by the following formula:

$$Y\;(\mathrm{yield})=\frac{\mathrm{weight}\;\mathrm{of}\;\mathrm{sample}}{\mathrm{weight}\;\mathrm{of}\;\mathrm{CCSK}\;(200\;g)}\times 100\%$$

### 2.5. Appearance

#### 2.5.1. Scanning Electron Microscopy (SEM)

According to Zhao et al. [21], the microstructures of the obtained CKE samples were observed. Briefly, the CKE samples were firstly sputter coated with approximately 10 nm of a conductive golden layer using an ion sputter coater (JFC-1600, JEOL Ltd., Akishima, Japan). Images were obtained using a cold field-emission scanning electron microscope (Regulus 8100, Hitachi Company, Tokyo, Japan) at an accelerating voltage of 3 kV and instrumental magnification 100× and 400×.

#### 2.5.2. Color Parameter Determination

The color parameters were determined using a colorimeter (LS171, Shenzhen Linshang Technology Co., Ltd., Shenzhen, China). The CKE samples were placed onto a white-background platform. The values obtained were described as L* (lightness), a* (degree of redness to greenness) and b* (degree of yellowness to blueness). The color difference (ΔE) between different CKE samples was calculated based on the CIEDE color-difference formula, according to Athira et al. [22].

### 2.6. Phytochemical Composition Determination

The following experiments were conducted in triplicate, respectively.

#### 2.6.1. Total Phenolic Content

The total phenolic contents were determined by ultraviolet (UV) spectrophotometrically [20] with slight modification. A detailed description is provided in Section S1.1.1 in the Supplementary Information.

#### 2.6.2. Total Flavonoid Content

The total flavonoid contents were determined by a previous method [10], with minor modification. A detailed description is provided in Section S1.1.2 in the Supplementary Information.

#### 2.6.3. Total Saponins Content

The vanillin–acetic acid method was employed to determine the total saponins content of the extracts [23]. A detailed description is provided in Section S1.1.3 in the Supplementary Information.

### 2.7. HPLC-ESI-QTOF-MS^2^ Analysis

According to Yan, Gao et al. [24], individual phenolic compounds were analyzed using an Agilent 1290 (Agilent, Waldbornn, Germany) equipped with an autosampler injector, a quaternary pump, a column oven and a diode array detector (DAD). Prior to analysis, the CKE samples at 1 mg/mL were dissolved by methanol (HPLC grade), and then filtered through a 0.22 μm organic phase needle filter (SCAA-104, ANPEL Laboratory Technology Inc., Shanghai, China). The chromatographic separation was accomplished using an Amethyst C18-H reverse-phase column (250 mm × 4.6 mm × 5 μm) (Sepax Technology Co., Ltd., DE, USA) at 25 °C, with an injection volume of 10 μL. The solvent system consisted of 0.1% (*v*/*v*) formic acid in ultrapure water (A) and acetonitrile (B). The gradient conditions were as follows: 5–95% B in 0–30 min; 95% B in 30–35 min; 95–5% B in 35–36 min; post-processing in 36–41 min. The flow rate was 0.8 mL/min, and the wavelength of UV detector was set at 280 nm. An AB SCIEX TripleTOF 5600 system (SCIEX, Foster City, CA, USA) was used for identification. The MS instrument was operated with electrospray source ionization (ESI) in negative mode. The major parameters were set as follows: ion spray voltage, −4500 V; source temperature, 550 °C; curtain gas, 40 psi; ion source gas 1, 50 psi; ion source gas 2, 50 psi; collision energy, −10 eV; and scan range, 100–1500 Da. Data acquisition and processing were performed with SCIEX OS software (Analyst^®^ TF 1.6 Software). Online databases, including MassBank (https://massbank.jp/, (accessed on 10 December 2023)), and SciFinder (https://scifinder-n.cas.org/, (accessed on 10 December 2023)), were used to analyze the MS^n^ data. The proportions of tentatively identified compounds were calculated by the area normalization method.

### 2.8. Identification of Volatile Compounds

The volatile compounds in the samples were extracted by headspace solid-phase microextraction (HS-SPME), coupled with a 50/30 µm divinylbenzene/carboxen/polydimethylsiloxane (DVB/CAR/PDMS) fiber, then analyzed according to He, Wu, and Yu [25]. Samples (1 mL, 5 mg/mL), dissolved in methanol, were transferred into 15 mL static headspace glass bottles, and then incubated at 50 °C for 20 min before the SPME fiber was inserted into the tube. After absorption for 30 min, the fiber was inserted into the injection port of the GC-MS and desorbed at 250 °C for 4 min.

The operating conditions of the Agilent 7890B gas chromatograph equipped with an MSD 5977 A mass spectrometer were as follows. A nonpolar fused quartz capillary column HP-5MS (30 m × 0.25 mm, 0.25 µm, Agilent, Santa Clara, CA, USA) was used for separation, with helium as a carrier gas at a flow rate of 1.8 mL/min. The front injection temperature was set to 250 °C. The oven temperature was programmed as follows: initial temperature of 40 °C held for 3 min, then increased from 40 °C to 240 °C at a rate of 5 °C/min and held for 5 min. The ionization was performed in EI. The ion source temperature, quadrupole temperature and auxiliary heater temperature were 230 °C, 150 °C and 250 °C, respectively.

According to Zhu et al. [8], the acquired mass spectrograms were compared with spectra in the National Institute of Standards and Technology Library (NIST 14), among which those with a positive and negative matching degree greater than 80 were retained. The proportions of volatile compounds were calculated by the area normalization method. Triplicated experiments were performed for each sample.

### 2.9. Measurement of In Vitro Antioxidant Activities

The following experiments were conducted in triplicate, respectively.

#### 2.9.1. DPPH Radical Scavenging Activity

The DPPH radical scavenging activity of the samples was assessed by the method of Zhao et al. [21] with some modifications. A detailed description is provided in Section S1.2.1 in the Supplementary Information.

#### 2.9.2. ABTS Radical Scavenging Activity

The ABTS radical scavenging activity of the samples was quantified following a previous method [26] with minor modifications. A detailed description is provided in Section S1.2.2 in the Supplementary Information.

#### 2.9.3. Ferric Reducing Antioxidant Power (FRAP)

A slightly revised FRAP assay was conducted according to a previous report [27]. A detailed description is provided in Section S1.2.3 in the Supplementary Information.

#### 2.9.4. Cupric Ion Reducing Activity (CUPRAC)

The CUPRAC assay was performed as previously described [28], with necessary modifications. A detailed description is provided in Section S1.2.4 in the Supplementary Information.

### 2.10. Identification of Volatile Compounds

#### 2.10.1. Emulsion Preparation

The emulsion was prepared according to a previous study [29]. A detailed description is provided in Section S1.3.1 in the Supplementary Information.

#### 2.10.2. Appearance Analysis

The appearance analysis was according to Khoozani et al. [30]. A detailed description is provided in Section S1.3.2 in the Supplementary Information.

#### 2.10.3. Measurement of Lipid Peroxide Value (POV)

The POV was determined according to Maqsood and Benjakul [31] with necessary modifications. A detailed description is provided in Section S1.3.3 in the Supplementary Information.

#### 2.10.4. Measurement of 2-Thiobabituric Acid-Reactive Substances (TBARS) Content

The TBARS contents were measured as described previously [32]. A detailed description is provided in Section S1.3.4 in the Supplementary Information.

#### 2.10.5. Measurement of Droplet Size and Zeta Potential

The particle size and zeta potential were measured according to Yan, Zhang et al. [33]. A detailed description is provided in Section S1.3.5 in the Supplementary Information.

### 2.11. Statistical Analysis

Results were expressed as mean ± standard deviation (SD) (*n* = 3). Data were analyzed by one-way ANOVA, followed by an F test with SPSS 26.0 software (SPSS, Inc., Chicago, IL, USA). Differences with a *p* value of less than 0.05 were considered statistically significant. The principal component analysis of the volatile compounds in the CKE samples was conducted using OriginPro 2024 (OriginLab Corporation, Northampton, MA, USA).

## 3. Results and Discussion

### 3.1. Extraction Efficiency

As shown in Table 1, the yield of CKE extracted by the SCFE method was the highest (1.83%, relative to CCSK) among the four different methods. The result may be explained by the high density, diffusivity and low viscosity of the sub-critical fluid, thereby increasing the internal mass transfer rate of the SCFE method and promoting the separation of oils and other bioactive substances in CCSK. Additionally, the mild temperature and pressure can also contribute to reducing the extraction time and degradation of the CKE [34]. In terms of CKE-AE, the yield was higher than those of CKE-SE and CKE-CP, which may be explained by mild operational conditions and the homogeneous medium that the colloid grinder provided, resulting in a more complete pulverization of CCSK [35].

### 3.2. Appearance

**Figure 1:**
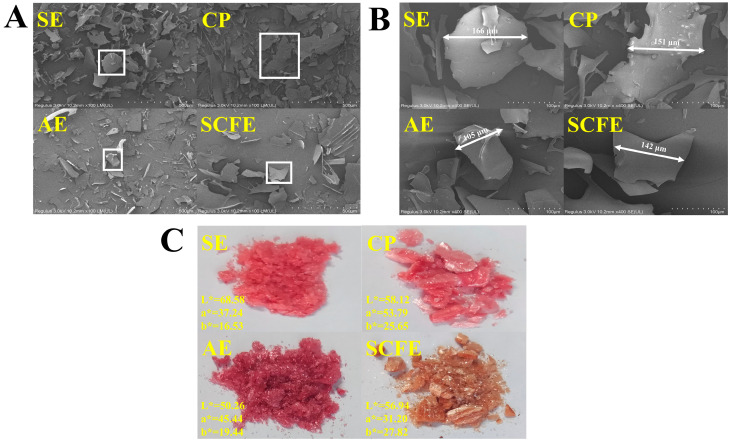
Scanning electron microscopies (SEM) (100×) (**A**), SEM (400×) (**B**), and appearance and color (**C**) of CKE samples.

As shown in Figure 1A, the four CKE samples showed typical lyophilizate-characteristic irregularly shaped particles and a broken glass structure of diverse size. The side length of the majority of the sample fragments was distributed from 100 μm to 200 μm (Figure 1B). As shown in Figure 1C, the four CKEs showed a typical flocculent structure. Additionally, as shown in Table 2, the color differences among the four CKE samples were all more than 5, indicating that the color differences were significant, and they can be perceived by humans [36]. On one hand, the color of CKE can be influenced by the oxidation degree and polymerization degree of polyphenol, which are commonly caused by different pretreatments [37]. For instance, in the presence of dissolved oxygen, flavanol can react with another flavanol molecule or with an anthocyanin, thereby forming flavanol–flavanol or flavanol–anthocyanin oligomers, changing the color of extract [38]. On the other hand, the color parameters of the samples can also be influenced by the profile of bioactive substances, especially that of polyphenols. Generally, high amounts of polyphenols and their derivatives can darken the color of the extracts [39]. Color parameters are vital factors for the acceptability of consumers in food choice [40]; thus, the CKE samples in diverse colors can meet different food production needs.

### 3.3. Bioactive Compounds

Bioactive compounds, including polyphenols, flavonoids and saponins, are widely considered as important constituents in plants due to their antioxidant and anti-inflammatory abilities [7]. As shown in Table 3, the contents of total phenolic and flavonoid equivalents (370.76 ± 1.59 mg GAE/g dw and 337.90 ± 5.68 mg RE/g dw, respectively) of CKE-SCFE were the highest among the samples processed, indicating that the bioactive substances were well protected in the oil extraction process using SCFE, due to its closed environment. Meanwhile, the total saponin content remained low, no matter what method was applied. The contents of total phenolic, flavonoid and saponin in CCSK were reported to be 122.90 ± 0.87 mg GAE/g dw, 196.92 ± 1.42 mg RE/g dw, and 127.14 ± 1.62 mg Rb1E/g dw, respectively [7], significantly different from this study. This difference may be explained by the high selectivity to phenolic compounds and flavonoids of AB-8 macroporous resin [41].

### 3.4. UHPLC-ESI-QTOF-MS^2^

**Figure 2:**
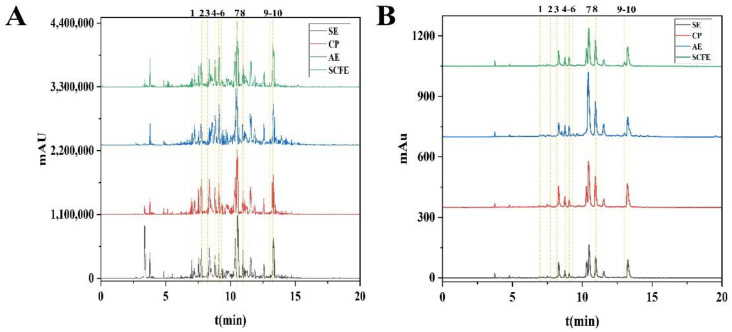
Total ion chromatogram (TIC) in negative ion mode (**A**) and ultra-performance liquid chromatography (UPLC) at 280 nm (**B**) of CKE samples.

The bioactive substances in the CKE were analyzed by UHPLC-ESI-QTOF-MS^2^ (Figure 2, Table 4 and Figure S1). A total of 10 compounds were tentatively identified by comparing their MS/MS spectra with the online databases, including MassBank (https://massbank.jp/, (accessed on 10 December 2023)), and SciFinder (https://scifinder-n.cas.org/, (accessed on 10 December 2023)). There were three flavonoids (peaks 1, 2 and 6), three phenolic acids (peaks 3, 4 and 5) and four alkaloids (peaks 7, 8, 9 and 10). Moreover, four unknown compounds were preliminarily identified Figure S2.

Peak 1 contained the molecular ion [M-H]- at *m*/*z* 477.1626 with fragments at *m/z* 179.0568, 221.0678 and 315.1101. The MS^2^ spectra presented a typical glucoside fragment (Δ[M-H]- = 162). Therefore, the compound was tentatively identified as isorhamnetin-3-O-β-D-glucoside. Peak 3 contained the molecular ion [M-H]- at *m*/*z* 341.0882 with fragments at *m*/*z* 135.0450, 179.0349 and 326.0873. The compound was tentatively identified as caffeic acid 4-O-glucoside (CAS number: 17093-82-2). Caffeic acid 4-O-glucoside can be found in *Plantago asiatica* L. and kiwi fruits; additionally, this compound showed a DPPH radical and O_2_^−^· radical scavenging capacity equivalent to approximately 18% and 25% of ascorbic acid, and showed inhibitive effects against neuroinflammatory-related factors (IKKβ and P38-α) [42].

Peak 2 contained the molecular ion [M-H]- at *m*/*z* 923.3467, with fragments at *m*/*z* 315.1090, 461.1683 and 631.2275. The MS^2^ spectra presented typical dimer fragments ([M-H]- = 461.1683 and [M-H]- = 923.3467). Therefore, the compound was tentatively identified as 3-[(6-deoxy-α-L-mannopyranosyl)oxy]-5,7-dihydroxy-2-(4′-methoxy)-6,7,8-trihydroxy-4H-1-benzopyran-4-one dimer. The compound has not been reported yet, and the compound name was based on 4′,5,7-trihydroxy-6-methoxyflavone (CAS number: 1440359-38-5) and MassBank (MSBNK-RIKEN-PR100798). It was reported that 4′,5,7-trihydroxy-6-methoxyflavone was found in *L. canum* and *L. fucata*, and it showed a better inhibitive effect against *M. luteus* than standard antibiotic ampicillin [43].

Peak 4 contained the molecular ion [M-H]- at *m*/*z* 711.2209, with fragments at *m*/*z* 193.0505, 355.1044 and 401.1102. The MS^2^ spectra presented typical glucoside and dirhamnoside fragments (Δ[M-H]- = 162 and Δ[M-H]- = 310). Therefore, the compound was tentatively identified as 5-(3,5-dihydroxy-1-(2′-carboxyl)-butyl)-phenyl β-D-glucopyranosiduronic acid dirhamnoside. The compound has not been reported yet, and its name was based on sinapoyl-ester-glucoside (CAS number: 1595079-71-2 and 136485-71-7). It was reported that sinapoyl-ester-glucoside was found in *Arabidopsis thaliana* and *Capsicum annuum* L., and its IC_50_ value of DPPH free radical scavenging capacity was 50.8 ± 2.36 μM [44].

Peak 5 contained the molecular ion [M-H]- at *m*/*z* 771.2403, with fragments at *m*/*z* 223.0606, 385.1142, 431.1201 and 589.2062. The MS^2^ spectra presented typical dimer fragments ([M-H]- = 385.1142 and [M-H]- = 771.2403). Therefore, the compound was tentatively identified as sinapoylglucose dimer. The compound has not been reported yet, and its name was based on (CAS number: 78185-48-5). Sinapoyl glucose was found in *C. limon* and *B. napus*, and its DPPH radical scavenging capacity was higher than 60.05% of Trolox equivalent [45].

Peak 6 contained the molecular ion [M-H]- at *m*/*z* 341.0871, with fragments at 238.8910, 293.1234 and 326.1390. The compound was tentatively identified as 7,3′,4′,5′-tetramethoxyflavone. Its chemical structure was tentatively identified via SciFinder (CAS number 855-97-0) and MassBank (MSBNK-BS-BS003507). This compound was found in *S. argentea*, and it showed in vitro anthelmintic activity [46].

Peak 7 contained the molecular ion [M-H]- at *m*/*z* 1409.6588, with fragments at 340.1563, 386.1617, 727.3298 and 1068.4936. The MS^2^ spectra presented typical tetramer fragments (Δ[M-H]- = 341) with a carboxyl group (Δ[M-H]- = 46). It was tentatively identified as isocorydine hydrochloride tetramer. Peak 8 contained the molecular ion [M-H]- at *m*/*z* 663.1973 with 340.1568 and 386.1618. The MS^2^ spectra exhibited a typical diglucoside fragment (Δ[M-H]- = 323). Its chemical structure was tentatively identified as isocorydine hydrochloride diglucoside, based on CAS number 475-67-2 and MassBank (MSBNK-Washington_State_Univ-BML82241). Isocorydine was found in *A. corneri* and *D. longipedicellata*; its IC_50_ value of DPPH free radical scavenging capacity and ferric ion reducing antioxidant power were 229.85 ± 7.51 μM and 0.79 ± 0.04 μM; it was reported that isocorydine significantly decreased the number of side population cells in hepatocellular carcinoma cell lines [47,48].

Peak 9 contained the molecular ion [M-H]- at *m*/*z* 206.0825, with fragments at 119.0502 and 163.0404. The compound was tentatively identified as N-acetyl-L-phenylalanine. Its chemical structure was identified via matching on SciFinder (CAS number 2018-61-3) and MassBank (MSBNK-RIKEN-PR309403). This compound was found in *Portulaca oleracea* L.; it showed an anti-browning ability for fresh-cut potato slices during storage, and its IC50 value of superoxide dismutase scavenging capacity was calculated to 99.57 μM via molecular docking analysis [49,50].

Peak 10 contained molecular ion [M-H]- at *m*/*z* 338.1406, with fragments at 167.0513, 209.0611, 237.0574, 265.0520 and 323.1178. This compound was found in *P. somniferum*; it suppressed cancer cell proliferation and pro-inflammatory cytokine production [51,52].

As shown in Figure S2, four peaks were tentatively identified as dimers with retention times at 3.780 min, 7.550 min, 8.367 min and 10.371 min, respectively. The MS^2^ spectra presented typical dimer fragments ([M-H]- = 238.8924 and Δ[M-H]- = 238; [M-H]- = 315.1089 and [M-H]- = 631.2282; [M-H]- = 325.0924 and [M-H]- = 651.1973; [M-H]- = 567.2525 and [M-H]- = 1135.5142), but their chemical structures are not identified yet.

It was reported that pretreatments influenced the contents of bioactive substances in oilseeds [53]. As shown in Table 3, a high level of isocorydine hydrochloride tetramers were found in all four CKE samples, and the contents of alkaloids in the CKE samples were higher than phenolic compounds. However, it does not mean that alkaloids are major substances in CKE samples, since only less than half of the chemical structures of the total substances were tentatively identified. Moreover, the percentage of identified substances in CKE-SCFE was 47.58, which was the highest among the four CKE samples, followed by CKE-CP, CKE-SE and CKE-AE. The result was consistent with the yield of the CKE samples (Table 1), which further confirmed the protective effect of bioactive substances on CKE samples using the SCFE method.

### 3.5. Volatile Compounds Composition

**Figure 3:**
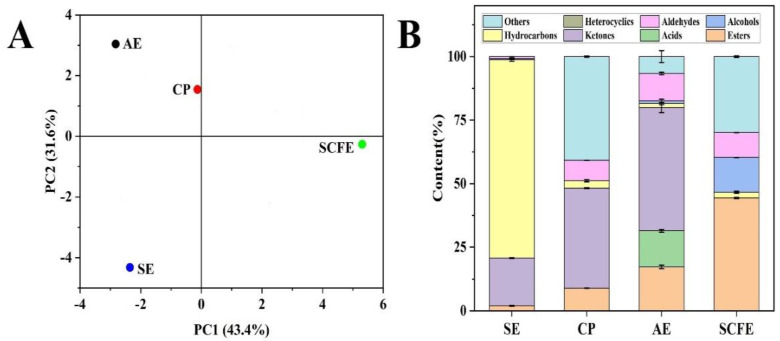
Principal component analysis (**A**) and content distribution (**B**) of volatile compounds in CKE samples.

Many phytochemicals are volatile compounds, thereby contributing to the characteristic aroma of the extracts [54]. As shown in Table 5, a total of 32 volatile compounds were identified, of which 9 were identified by CKE-SE, 12 by CKE-CP, 14 by CKE-AE and 18 by CKE-SCFE, respectively. The principal component analysis (PCA) of the volatile compounds in the four CKE samples is depicted in Figure 3A. The first and second principal components (PC1 and PC2), which included all the data information including volatile compounds contents, explained 43.4% and 31.6% of the total variations in clinical parameters, respectively. A significant separation of the pretreated CKE samples was observed. Only CKE-SCFE showed positive values in PC1; the other three CKE samples presented negative values in PC1. Meanwhile, CKE-AE and CKE-CP showed positive values in PC2, while CKE-SCFE and CKE-SE were negative in PC2. The results indicated that the volatile substances in the four CKE samples were significantly different. As shown in Figure 3B, the contents of volatile compounds in the four CKE samples varied significantly; the predominant substances in the samples were hydrocarbons (77.95 ± 0.47%) in CKE-SE, ketones (48.42 ± 1.97%) in CKE-AE, esters (44.37 ± 0.19%) in CKE-SCFE and other substances (40.86 ± 0.22%) in CKE-CP, respectively. To be specific, the predominant substances were 5-hydroxy-4-methyl-6-hepten-3-one (38.398 ± 0.209%) in CKE-CP, 2-methyl butane (77.774 ± 0.358%) in CKE-SE, 3-pentanone (48.413 ± 1.967%) in CKE-AE, and oxalic acid allyl isobutyl ester (26.415 ± 0.092%) in CKE-SCFE, respectively. Ketones are formed by the β-oxidation of fatty acids [55], and the CCSK (rich in free fatty acid) was in full contact with the air in the colloid grinder, which might explain why the predominant volatile substances of CKE-AE were ketones. Additionally, esters are predominant substances in CKE-SCFE, which may be due to the full contact of the free fatty acids and alcohols of CCSK in closed containers, thereby generating esterification reactions [56]. The results showed that pretreatment methods significantly affected the composition of the volatile compounds, which was consistent with Wei et al. [57], who found that the processing technique affected the concentrations of volatile compounds. Therefore, the four CKE samples with different characteristic aromas may play different roles in industries.

### 3.6. Antioxidant Capacities

As shown in Table 6, the metal ion reducing capacity (expressed as FRAP and CUPRAC values) of the four CKE samples were significantly higher than their free radical scavenging capacity (expressed as DPPH and ABTS values), and the FRAP and CUPRAC values of the four CKE samples were higher than Trolox equivalent, indicating the excellent metal ion reducing capacity of the CKEs. It was reported that bioactive substances (especially flavonoids) show lipid antioxidant capacity through inhibiting metal ions from catalyzing the production of lipid hydroperoxides [58]. In addition, the ABTS value of each CKE sample was significantly higher than the DPPH value. This result was consistent with Zhang et al. [7], who found that the DPPH and ABTS values of crude CKE were 2.40 mmol TE/g dw and 7.85 mmol TE/g dw, respectively. It is worthwhile noting that the in vitro antioxidant activity of CKE-SCFE was the highest among the four samples. This may be due to the highest concentration of antioxidants (especially phenolic compounds) of CKE-SCFE among the four CKE samples [59]. The CKE samples, as residues and by-products of CCSK production, showed excellent in vitro antioxidant capacities (including free radical scavenging capacity and metal ion chelating ability) due to their lipophilic and hydrophilic phytochemicals [60], including Peak 3, Peak 4, Peak 5, Peak 7 and Peak 8 described in Section 3.4, indicating that they may have great application potential in the food industry.

### 3.7. Lipid Antioxidant Assay

Perilla oil has nutritional functions due to its richness in ω-3 unsaturated fatty acid, especially alpha linolenic acid (ALA), but ALA-rich oil is susceptible to autoxidation and thermal oxidation [61]. Additionally, oil-in-water (O/W) emulsions are widely used in the food industry because they are compatible with other aqueous-based systems. However, O/W emulsions are susceptible to spoilage and/or rancidity, as a result of their internal thermodynamically unstable properties and lipid oxidation [62]. Therefore, perilla oil-based emulsions are ideal systems for amplifying the oxidation degree of lipids, thereby better evaluating the anti-lipid peroxidation capacities of bioactive substances within a reasonable experimental period.

#### 3.7.1. Emulsion Appearance

**Figure 4:**
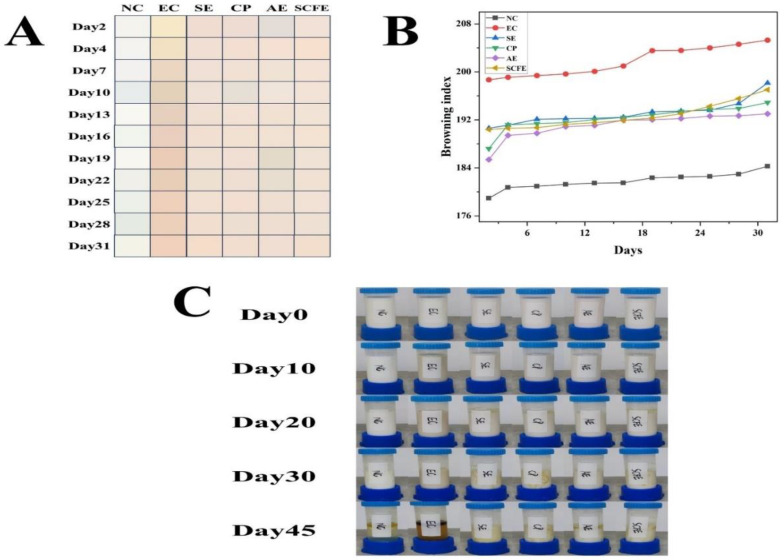
Changes in color (**A**), browning index (**B**), and appearance (**C**) of emulsions added with different CKE samples.

The changes of color in emulsions added with different samples at different times are shown in Figure 4A and Table S1. The values of a* and b* of the NC group showed a downward and an upward trend, respectively. The L* value gradually decreased and the a* value increased in the EC group, while the other four CKE groups (the SE, CP, AE and SCFE groups) maintained a stable trend of chrominance change. As shown in Figure 4B and Table S1, the browning indexes of the CKE groups were between the EC group and NC group. The composition and concentration of bioactive substances determine the oxidation and browning degrees, as well as the flavor of emulsions, thereby affecting the palatability and acceptability of food products [40,63]. The results showed that CKE had a better capacity for inhibiting the browning of the emulsion than epicatechin. As shown in Figure 4C, all of the groups (except the SE and CP groups on day 30) did not show obvious stratification or oiling off in the initial 30-day experimental period. It was found that the oil phase and water phase were completely stratified in the NC and EC groups on day 45, while the CKE groups still retained a basic emulsion form, indicating that CKE may have more potential emulsion stability and thermal stability than synthetic bioactive substances [64].

#### 3.7.2. POV

**Figure 5:**
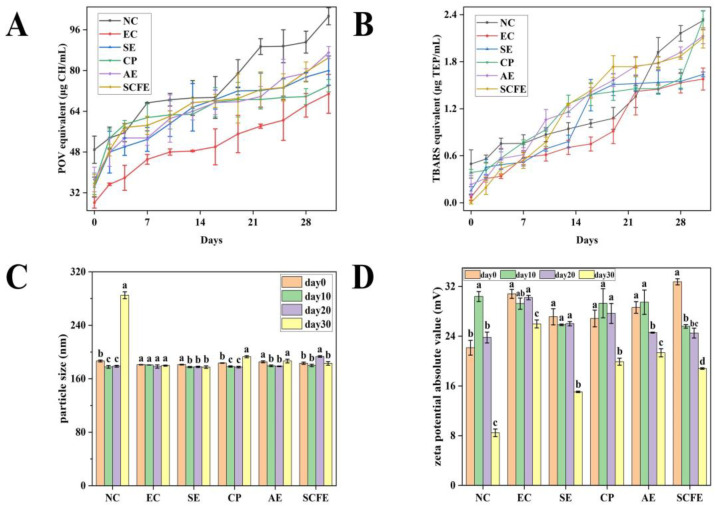
Changes in peroxide values (POV) (**A**), 2-thiobabituric acid-reactive substances (TBARS) values (**B**), particle sizes (**C**) and zeta potentials (**D**) of emulsions added with different samples. Data are expressed as mean ± SD. The marked different letters in the same group in (**C**,**D**) indicate significant difference (*p* < 0.05).

The undesirable flavors and colors of emulsions are commonly produced from hydroperoxides, which are widely considered as the first peroxide of lipids, thereby deteriorating the quality and safety of products [65,66,67]. Thus, it is essential to determine the POV of different emulsion samples. As shown in Figure 5A, the POVs of the NC and EC groups reached maximum values of 101.31 ± 3.36 μg CH/mL and 70.85 ± 7.62 μg CH/mL after a 30-day incubation, respectively. Meanwhile, the final POVs of the SE, CP, AE and SCFE groups were 80.01 ± 5.72 μg CH/mL, 74.05 ± 2.44 μg CH/mL, 87.05 ± 2.39 μg CH/mL and 85.03 ± 0.00 μg CH/mL, respectively. The centrifuge tubes were kept at a constant temperature in the dark during the 30-day experimental period; thus, light may not be a primary factor to trigger oxidation. Instead, lipid oxidation between the unsaturated components of the perilla oil and oxygen in emulsions is commonly caused by the radicals existing in the aqueous phase [68]. It was reported that antioxidants inhibited lipid oxidation via donating hydrogen atoms to lipid radicals [31]. Overall, the POV values of the CP and SE groups were lower than that of AE and SCFE groups. The CP and SE groups showed no significant difference with the EC group, while the POV values in the AE and SCFE groups were significantly higher than the EC group. The four CKE groups had the inhibitive capacity of hydroperoxides, with similar POV values.

#### 3.7.3. TBARS Value

TBARS values measure the generation of secondary oxidation products from hydroperoxides or the oxidized polyunsaturated fatty acids of perilla oil [67,69]. As shown in Figure 5B, the TBARS value of the NC group increased from 0.49 ± 0.18 μg TEP/mL to 2.32 ± 0.12 μg TEP/mL; the peak TBARS values of the CP, AE and SCFE groups were 2.34 ± 0.11 μg TEP/mL, 2.13 ± 0.10 μg TEP/mL and 2.10 ± 0.13 μg TEP/mL, respectively. Meanwhile, the final TBARS values of the EC and SE groups were similar (1.57 ± 0.14 μg TEP/mL and 1.64 ± 0.03 μg TEP/mL, respectively). A gradually increasing trend in TBARS values was observed in this study. Taghvaei et al. [64] found a general increase trend with a slight dip during the experimental period. This may be because the emulsion systems in this study did not completely trigger the further oxidation of hydroperoxides or polyunsaturated fatty acids of lipids, thereby failing to cause secondary autoxidation or form carboxylic acids. Overall, CKE-SE exhibited a better inhibitive capacity of secondary oxidation products among the four CKE samples.

#### 3.7.4. Droplet Size and Zeta Potential

The measurement of the droplet size and zeta potential of emulsion samples can provide information on the dispersion mechanism and colloidal stability of bioactive substances [33]. As shown in Figure 5C, the droplet size of the NC group significantly increased in the 30-day experimental period, while the other five groups did not show an obvious change. The results may stem from the predominant existence of polyphenols with at least two phenyl rings in CKEs, as they have the capacity to provide plenty of π-electrons, thereby maintaining emulsion stability via promoting the electrostatic repulsion of lipid droplets and restraining depletion flocculation [29,70]. On the other hand, the amphiphilic molecules of polyphenol compounds contributed to increasing the affinity to the oil–water interface [71]. As shown in Figure 5D, the zeta potential absolute values of all the six emulsion samples fluctuated within the first 20-day period. However, they significantly decreased in the last ten experimental days, and the zeta potential absolute value of the NC group was obviously lower than those of the other five groups. The significant decrease in zeta potential absolute value in the NC group may be explained by emulsion rancidity, while antioxidant substances in other groups have the capacity to scavenge free radicals, such as hydrogen peroxide [72], which can be confirmed by the significantly increased POV value in the NC group after day 20 (Figure 5A). Moreover, Tween 80, used as a small molecular surfactant in this study, has the ability to adsorb on the water–oil interface, then play the role of emulsifier [73]. However, whether there is a synergistic effect between CKE and Tween 80 remains to be investigated. Overall, the four CKE groups had the capacity of maintaining the stability of the emulsion system, although their zeta potentials were lower than EC.

## 4. Conclusions

The results showed that the CKE samples were rich in phenolic compounds and alkaloids, and the phytochemicals contents varied in different CKEs. The extraction yield, bioactive substances content, free radical scavenging capacity and metal ion chelating ability of CKE-SCFE were better than the other CKE samples. All the CKE samples showed a lipid emulsion oxidation and delamination inhibitive capacity, with CKE-SE showing the best lipid emulsion oxidation inhibitive capacity. Moreover, the volatile substances composition in the CKE samples was greatly influenced by the pretreatment method. The study laid a theoretical foundation for the further utilization of CKE in the food and pharmaceutical industries. Subsequent research could investigate the process parameters in CKE scale-up production and the biological activities of CKE in real products.

## Figures and Tables

**Table 1:** Weight and yield of CKE samples pretreated by four different methods.

	SE	AE	CP	SCFE
Purified extract weight (g)	1.823 ± 0.007 ^c^	2.613 ± 0.097 ^b^	1.592 ± 0.030 ^d^	3.654 ± 0.121 ^a^
Purified extract yield (%)	0.91 ± 0.00 ^c^	1.31 ± 0.05 ^b^	0.80 ± 0.02 ^d^	1.83 ± 0.06 ^a^

CKE: *C. camphora* seed kernel extract; SE, solvent extraction method; AE, aqueous extraction method; CP, cold pressing method; SCFE, sub-critical fluid extraction method. The marked different letters in the same row indicate a significant difference (*p* < 0.05).

**Table 2:** Color difference between CKE samples pretreated by four different methods.

	SE	CP	AE	SCFE
SE	-			
CP	10.55	-		
AE	16.50	8.16	-	
SCFE	12.11	8.97	10.36	-

CKE: *C. camphora* seed kernel extract; SE, solvent extraction method; AE, aqueous extraction method; CP, cold pressing method; SCFE, sub-critical fluid extraction method.

**Table 3:** Contents of total phenols, flavonoids and saponins in CKE samples (mean ± SD, *n* = 3).

	SE	AE	CP	SCFE
Total phenols content (mg GAE/g dw)	346.88 ± 3.30 ^c^	347.13 ± 4.13 ^c^	359.25 ± 0.56 ^b^	370.76 ± 1.59 ^a^
Total flavonoid content (mg RE/g dw)	303.06 ± 7.04 ^c^	370.36 ± 8.06 ^a^	350.12 ± 16.17 ^a^	337.90 ± 5.68 ^ab^
Total saponin content (mg Rb1E/g dw)	49.26 ± 2.53 ^a^	29.50 ± 2.22 ^c^	15.19 ± 2.23 ^d^	37.61 ± 2.29 ^b^

CKE: *C. camphora* seed kernel extract; SE, solvent extraction method; AE, aqueous extraction method; CP, cold pressing method; SCFE, sub-critical fluid extraction method; GAE, gallic acid equivalent; RE, rutin equivalent; Rb1E: ginsenoside-Rb1 equivalent; dw, dry weight. The marked different letters in the same row indicate significant difference (*p* < 0.05).

**Table 4:** Tentative identification of bioactive compounds in CKE using UHPLC-ESI-QTOF-MS^n^.

Peaks	Rt (min)	Error (ppm)	Type of Compound	[M-H]- (*m*/*z*)	MS/MS (*m*/*z*)	Peak Area Percentage				Tentative Identification	Reference
						SE	CP	AE	SCFE		
1	7.017	0.4	Flavonoid	477.1626	179.0568, 221.0678, 315.1101	1.10	1.39	0.92	0.45	Isorhamnetin-3-O-β-D-glucoside	[24]
2	7.754	0.5	Flavonoid	923.3467	315.1090, 461.1683, 631.2275	4.69	6.49	3.45	4.75	3-[(6-deoxy-α-L-mannopyranosyl)oxy]-5,7-dihydroxy-2-(4′-methoxy)-6,7,8-trihydroxy-4H-1-benzopyran-4-one dimers	-
3	8.229	0.4	Phenolic acid	341.0882	135.0450, 179.0349, 326.0873	0.65	0.50	0.73	0.55	Caffeic acid 4-O-glucoside	[24]
4	8.809	0.9	Phenolic acid	711.2209	193.0505, 355.1044, 401.1102	4.02	4.47	5.15	4.79	5-(3,5-dihydroxy-1-(2′-carboxyl)-butyl)-phenyl β-D-glucopyranosiduronic acid dirhamnoside	-
5	9.114	0.8	Phenolic acid	771.2403	223.0606, 385.1142, 431.1201, 589.2062	3.19	5.09	5.77	7.57	Sinapoylglucose dimers	[10,24]
6	9.325	1.1	Flavonoid	341.0871	238.8910, 293.1234, 326.1390	1.62	2.35	1.43	2.05	7,3′,4′,5′-tetramethoxyflavone	-
7	10.542	0.0	Alkaloid	1409.6588	340.1563, 386.1617, 727.3298, 1068.4936	13.90	14.53	13.89	13.30	Isocorydine hydrochloride tetramers	-
8	10.977	1.2	Alkaloid	663.1973	340.1568, 386.1618	2.80	2.85	3.08	3.94	Isocorydine hydrochloride diglucoside	-
9	13.085	1.2	Alkaloid	206.0825	119.0502, 163.0404	0.42	0.24	0.35	0.18	N-acetyl-L-phenylalanine	-
10	13.313	−0.1	Alkaloid	338.1406	167.0513, 209.0611, 237.0574, 265.0520, 323.1178	9.82	8.62	7.13	9.99	Papaverine	[7,9,10]
Phenolic compounds						15.28	20.29	17.45	20.17		
Alkaloids						26.94	26.24	24.44	27.41		
Total						42.22	46.53	41.89	47.58		

CKE: *C. camphora* seed kernel extract; SE, solvent extraction method; AE, aqueous extraction method; CP, cold pressing method; SCFE, sub-critical fluid extraction method; Rt, retention time.

**Table 5:** Volatile compounds composition of CKE pretreated by different processing methods.

No.	Groups	Compounds	Formula	*m*/*z*	CP	SE	AE	SCFE
1	Esters	1,3-Benzodioxole-5-carboxylic acid methyl ester	C_9_H_8_O_4_	149	nd	nd	0.022 ± 0.000	nd
2		2-Propyn-1-ol acetate	C_5_H_6_O_2_	98	nd	nd	nd	10.533 ± 0.035
3		Acetic acid hydroxy-ethyl ester	C_4_H_8_O_3_	104	nd	nd	nd	7.306 ± 0.026
4		Benzoic acid 4-methoxy-methyl ester	C_9_H_10_O_3_	166	nd	nd	nd	0.045 ± 0.000
5		Decanoic acid methyl ester	C_11_H_22_O_2_	186	0.029 ± 0.016 ^ab^	0.120 ± 0.088 ^a^	0.035 ± 0.017 ^ab^	0.034 ± 0.021 ^ab^
6		Dodecanoic acid methyl ester	C_13_H_26_O_2_	214	0.025 ± 0.007 ^b^	0.434 ± 0.020 ^a^	0.024 ± 0.002 ^b^	0.017 ± 0.007 ^bc^
7		isocyanato-methane	C_2_H_3_NO	56.1	nd	1.393 ± 0.007	nd	nd
8		Oxalic acid allyl isobutyl ester	C_9_H_14_O_4_	186	8.792 ± 0.046 ^b^	nd	6.673 ± 0.271 ^c^	26.415 ± 0.092 ^a^
9		Phthalic acid 3,5-dimethylphenyl 4-formylphenyl ester	C_23_H_18_O_5_	374	nd	nd	nd	0.016 ± 0.005
10		p-Toluic acid 4-cyanophenyl ester	C_15_H_11_NO_2_	237	nd	0.003 ± 0.001	nd	nd
11		α-Amino-γ-butyrolactone	C_4_H_7_NO_2_	101	nd	nd	10.497 ± 0.427	nd
12	Acids	Propanoic acid anhydride	C_6_H_10_O_3_	130	nd	nd	14.222 ± 0.578	nd
13	Ketones	3-Pentanone	C_5_H_10_O	86	nd	18.781 ± 0.086 ^b^	48.413 ± 1.967 ^a^	nd
14		5-Hydroxy-4-methyl-6-hepten-3-one	C_8_H_14_O_2_	142	38.398 ± 0.209	nd	nd	nd
15		2-(formyloxy)-1-phenyl-ethanone	C_9_H_8_O_3_	164	0.002 ± 0.000 ^b^	0.006 ± 0.001 ^a^	0.003 ± 0.000 ^a^	0.001 ± 0.000 ^c^
16	Hydrocarbons	1,3-Dioxolane	C_3_H_6_O_2_	73	nd	nd	0.004 ± 0.000	nd
17		2-methyl butane	C_5_H_12_	57.1	nd	77.774 ± 0.358	nd	nd
18		Methyl-cyclopentane	C_6_H_12_	84	1.792 ± 0.450 ^a^	nd	1.150 ± 0.401 ^a^	0.957 ± 0.002 ^ab^
19		Ethane	C_2_H_6_	30.1	nd	nd	nd	1.283 ± 0.424
20		Ethylene	C_2_H_4_	28	0.905 ± 0.005	nd	nd	nd
21		3-methyl-pentane	C_6_H_14_	86	0.202 ± 0.001 ^b^	nd	0.424 ± 0.017 ^a^	nd
22		2-methyl-1-nitro-propane	C_4_H_9_NO_2_	103	nd	0.181 ± 0.111	nd	nd
23	Alcohols	5,5-dioxide 3,5-Dithiahexanol	C_4_H_10_O_3_S_2_	170	nd	0.485 ± 0.323 ^b^	1.036 ± 0.715 ^a^	nd
24		α-[1-(ethylmethylamino)ethyl]-benzenemethanol	C_12_H_19_NO	193	nd	nd	nd	13.702 ± 0.047
25	Aldehydes	2-Propenal	C_3_H_4_O	56.1	nd	0.823 ± 0.004 ^b^	nd	1.132 ± 0.005 ^a^
26		2,2-dimethyl-propanal	C_5_H_10_O	86	7.998 ± 0.043 ^c^	nd	10.824 ± 0.440 ^a^	8.709 ± 0.031 ^b^
27	Heterocyclics	5-Hydroxy-7-methoxy-2-methyl-3-phenyl-4-chromenone	C_17_H_14_O_4_	282	nd	nd	nd	0.003 ± 0.000
28		5-Methyl-2-(2-methyl-2-tetrahydrofuryl)tetrahydrofuran	C_10_H_18_O_2_	170	nd	nd	nd	0.021 ± 0.000
29	Others	Butanenitrile	C_4_H_7_N	69	nd	nd	nd	7.886 ± 0.028
30		Bis(1,1-dimethylethyl)-diazene	C_8_H_18_N_2_	142	13.289 ± 0.070	nd	nd	nd
31		Hydrazine	H_4_N_2_	32	27.566 ± 0.151 ^a^	nd	nd	21.940 ± 0.278 ^b^
32		Hydrogen azide	HN_3_	43.1	nd	nd	6.672 ± 2.349	nd

CKE: *C. camphora* seed kernel extract; SE, solvent extraction method; AE, aqueous extraction method; CP, cold pressing method; SCFE, sub-critical fluid extraction method. The marked different letters in the same row indicate significant difference (*p* < 0.05).

**Table 6:** Antioxidant capacities of CKE samples (mean ± SD, *n* = 3).

	SE	AE	CP	SCFE
DPPH radical (mg Trolox/g dw)	200.58 ± 1.82 ^a^	203.22 ± 2.82 ^a^	158.19 ± 10.97 ^b^	200.00 ± 1.52 ^a^
ABTS radical (mg Trolox/g dw)	607.95 ± 5.77 ^c^	618.90 ± 11.54 ^c^	656.52 ± 14.09 ^b^	702.24 ± 19.18 ^a^
FRAP (mg Trolox/g dw)	1296.33 ± 45.81 ^a^	1326.33 ± 49.85 ^a^	1336.08 ± 48.87 ^a^	1328.38 ± 47.04 ^a^
CUPRAC (mg Trolox/g dw)	1205.28 ± 7.99 ^c^	1225.78 ± 14.42 ^c^	1418.12 ± 2.99 ^b^	1455.41 ± 16.94 ^a^

CKE: *C. camphora* seed kernel extract; SE, solvent extraction method; AE, aqueous extraction method; CP, cold pressing method; SCFE, sub-critical fluid extraction method; DPPH, 1,1-diphenyl-2-picrylhydrazyl; ABTS, 2,2′-azinobis-(3-ethylbenzthiazoline-6-sulphonate); FRAP, ferric reducing antioxidant power; CUPRAC, cupric ion reducing activity; dw, dry weight. The marked different letters in the same row indicate significant difference (*p* < 0.05).

## Data Availability

The original contributions presented in the study are included in the article/Supplementary Materials, further inquiries can be directed to the corresponding authors.

## Supplementary Materials

The following supporting information can be downloaded at: https://www.mdpi.com/article/10.3390/foods13132064/s1, Figure S1: MS^2^ spectra and possible fragmentation patterns of tentatively identified compounds in CKE; Figure S2: MS^2^ spectra of four potential dimers in CKE.
