# Supplementary material for: Effect of Pretreatments on the Chemical, Bioactive and Physicochemical Properties of Cinnamomum camphora Seed Kernel Extracts
Source: Foods. 2024 Jun 28;13(13):2064. doi: 10.3390/foods13132064 (PMC11241286; doi:10.3390/foods13132064)
Supplement: Supplementary file 1 [file foods-13-02064-s001.zip › foods-3052326-supplementary.pdf]

foods

MDPI

Supplementary Information

S1. Methods

S1.1. Content determination

S1.1.1. Total phenolic content

Briefly, 200 μL of Folin–Ciocalteu reagent was mixed with the sample solution (1 mL, 0.5 mg/mL) and standard solution of garlic acid (1 mL, 0-0.25 mg/mL). Afterwards, Na_2CO_3 solution (2 mL, 7%) was added and oscillated thoroughly. The mixture was incubated at room temperature in the dark for 60 min. The final volume was completed to 4.8 mL with distilled water. The tubes were again incubated at room temperature in the dark for 90 min after vigorous stirring. The sample absorbances were read at 760 nm using a UV spectrophotometer. The total phenolic contents were expressed as milligrams of gallic acid equivalents (mg GAE/g extract dw).

S1.1.2. Total flavonoid content

Once sample solution (2.5 mL, 1 mg/mL) was prepared in the tubes; $NaNO_2$ (0.3 mL, 5%) was added and incubated at room temperature for 6 min. Then $Al(NO3)_3$ (0.3 mL, 17.6%) solution and NaOH (4.4 mL, 4%) were added, followed by incubation for 6 min and 12 min, respectively. Absorbances were measured at 510 nm. The results were expressed as milligram of rutin equivalents per gram of dry weight of sample (mg RE/g sample dw).

S1.1.3. Total saponins content

A nitrogen concentration was applied after the samples (200 μL, 1 mg/mL) were added to the tubes. Once the solution was thoroughly blown dry, a vanillin–acetic acid mixture (0.2 mL, 5:95 (m:m)) was added, followed by 0.8 mL perchloric acid. The mixture was incubated in a 60 °C thermostat water bath for 10 min and was then cooled down to room temperature in an ice-water bath. A 5 mL aliquot of acetic acid was added, and the tubes were oscillated. Then, the mixture was stored in the dark for 30 min, followed by absorbance measurement at 560 nm. Ginsenoside Rb1 was used as a standard, and the content of total saponins was expressed as milligram of ginsenoside Rb1 equivalents per gram of dry weight of sample (mg GE/g sample dw).

S1.2. In vitro antioxidant capacities

S1.2.1. Measurement of DPPH scavenging activity

To prepare different concentrations of Trolox solutions (2 mL, 0-0.02 mol/L), Trolox and 80% (v/v) ethanol were added. Sample solutions (2 mL) were mixed with a 2 mL 80% (v/v) ethanol solution of DPPH. Absorbances were measured at 517 nm after incubation at room temperature for 30 min, and the results were expressed as μmol of Trolox equivalent (TE) per gram of sample dry weight (μmol TE/g sample dw).

S1.2.2. Measurement of ABTS scavenging activity

ABTS (7 mM) and $K_2S_2O_8$ (2.45 mM) were dissolved in Milli-Q water in 10 mL volumetric flask, respectively. The ABTS stock solution was prepared by mixing the two solutions above and incubating them at room temperature in the dark for 12-16 h. Subsequently, ultra-pure water was used to acquire a working solution with an absorbance of 0.70 ± 0.02 at 734 nm. The working solution (3.9 mL) was then mixed with 0.1 mL sample solution. The absorbances were measured at 734 nm, and the results were calculated as μmol of Trolox equivalent (TE) per gram of sample dry weight (μmol TE/g sample dw).

S1.2.3. Ferric reducing antioxidant power

The FRAP working solution was freshly prepared by reacting several stock solutions with a ratio of 10:1:1 (v/v/v), which were 1) 10 mM TPTZ solution in 40 mM HCl, 2) 20 mM $FeCl_3$ solution, and 3) acetate buffer containing 0.31 g sodium acetate and 1.6 mL acetic acid. The FRAP solution was prewarmed at 37 °C before a 0.4 mL sample solution was added, followed by incubation in a thermostatic water bath at 37 °C for 10 min. Different concentrations (0-0.25 mol/L) of Trolox solutions were prepared with 80% (v/v) ethanol to obtain the calibration curve solutions. The absorbances of the mixture were read and recorded by a UV spectrophotometer at 593 nm. The results were expressed as μmol of Trolox equivalent (TE) per gram of sample dry weight (μmol TE/g sample dw).

S1.2.4. Cupric ion reducing activity

Three stock solutions were mixed to acquire the CUPRAC working solution, including an equal volume of 10 mM $CuCl_2$ solution, 7.5 mM neocuproine solution in ethanol and 1 M ammonium acetate buffer. Then, the abovementioned 3 mL of the CUPRAC working solution was added into each test tube with 0.7 mL sample solution. The corresponding volume of Milli-Q water was used to adjust the volume to 4.1 mL. The resulting mixtures were incubated at room temperature for 30 min, and the absorbances were measured at 450 nm. The results were expressed as μmol of Trolox equivalent (TE) per gram of sample dry weight (μmol TE/g sample dw).

S1.3. Lipid antioxidant assay

S1.3.1. Emulsion preparation

A considerable volume of perilla seeds were crushed by a pulverizer and then mixed with n-hexane at the ratio of 1:3 (m/v). The mixture was continuously stirred for 4 h and then separated by vacuum filtration, and the abovementioned process was repeated three times. The combined filtrate was concentrated by vacuum rotary evaporation to obtain the perilla oil.

The emulsion was composed of 10% perilla seed oil extract, 1% Tween 80 and 89% distilled water (m/m/m). The CKE pretreated by four different methods and epicatechin were added into five aliquots of the abovementioned untreated emulsion, respectively, with the same final concentration of 1 mg/mL (SE, CP, AE, SCFE and EC groups). Meanwhile, an additional aliquot containing emulsion only was set as a negative control (NC group). The mixtures were dispersed at 10,000 rpm by a high-speed disperser for 20 min to obtain perilla oil macroemulsions. After that, the abovementioned macroemulsions were passed through a high-pressure homogenizer at 40 MPa for 5 min for four consecutive cycles to obtain perilla oil nanoemulsions.

S1.3.2. Appearance analysis

The color parameters were measured from day 0 to day 28 at 3-day intervals using a colorimeter (LS171, Linshang Technology Co., Ltd., Shenzhen, China). The emulsion samples were pipetted and placed onto a glass slide against a black background. The values obtained were described with L* (lightness), a* (degree of redness to greenness) and b* (degree of yellowness to blueness). The browning index was calculated by the following formula:

$$\text{Browning index}=\frac{100\times(x-0.31)}{0.17}$$

$$x=\frac{a^{*}+1.75L^{*}}{5.645L^{*}+a^{*}-3.012b^{*}}$$

where L* represents lightness, a* represents the degree of redness to greenness, and b* represents the degree of yellowness to blueness.

S1.3.3. Measurement of lipid peroxide value (POV)

Emulsion samples (0.3 mL) were mixed with 1.5 mL of isooctane/isopropanol (3:1, v/v). The mixture was vigorously vortexed and then centrifuged at 8000 rpm for 5 min to separate it into two phases. The upper phase (0.2 mL) was pipetted and added to the test tube with 2 mL of freshly prepared ferrous sulfate solution (0.144 M) and 50 μL of 3.94 M ammonium thiocyanate solution. The vigorously vortexed mixtures were incubated at room temperature for 20 min prior to reading the absorbance at 510 nm. The POV was calculated using a standard curve of cumyl hydroperoxide (CH) and expressed as mg CH equivalents per g of oil. The experiment was conducted in triplicate.

S1.3.4. Measurement of 2-thiobabituric acid-reactive substances (TBARS) content

The 200 μL of upper layer described in Supplementary Information Section 1.3.3 was added into 100 μL of 2% (m/v) butylated hydroxytoluene (BHT) methanol solution. Then 2 mL of thiobarbituric acid (TBA) solution and 0.7 mL of deionized water were mixed in each test tube. The mixture was heated in boiling water for 10 min and cooled down to room temperature. Five minutes of centrifugation at 8000 rpm was applied. The absorbance of the supernatant was measured at 532 nm. All values were expressed as mg 1,1,3,3-tetraethoxypropane (TEP) equivalents per g of oil. The experiment was conducted in triplicate.

A TBA solution was prepared by mixing 0.45 g TBA, 18 g trichloroacetic acid and 100 mL of 0.25 M HCl (100 mL) solution, and then dissolved by stirring.

S1.3.5. Measurement of particle size and zeta potential

The abovementioned emulsion samples were diluted by distilled water, with the dilution ratio of 1:500 (v/v). The particle size and zeta potential were measured at 25 °C at 10-day intervals using a Zetasizer (Nano ZS90, Malvern Instruments Ltd., Worcestershire, UK). Triplicate analytical measurements were performed for each sample.

S2. Results

S2.1. Bioactive compounds

The total polyphenol contents in the CKE samples were calculated by the calibration equation (y = 0.0055x + 0.0174, R^2 = 0.9988) derived from the calibration curve of the gallic acid standard. The results were expressed as milligram of gallic acid equivalents per gram of dry weight sample (mg RE/g dw).

The total flavonoid contents in the CKE samples were calculated by the calibration equation (y = 0.0042x + 0.0165, R^2 = 0.9957) derived from the calibration curve of the rutin standard. The results were expressed as milligram of rutin equivalents per gram of dry weight sample (mg RE/g dw).

The total saponin contents in the CKE samples were calculated by the calibration equation (y = 0.0064x + 0.0317, R^2 = 0.9997) derived from the calibration curve of the ginsenoside-Rb1 standard. The results were expressed as milligram of ginsenoside-Rb1 equivalents per gram of dry weight sample (mg RE/g dw).

S2.2. Antioxidant capacities

The DPPH scavenging capacity (presented as DPPH value) in the CKE samples was calculated by the calibration equation (y = -0.0114x + 0.0269, R^2 = 0.997) derived from the calibration curve of the Trolox standard, and expressed as milligram of Trolox equivalents per gram of dry weight sample (mg Trolox/g dw).

The ABTS scavenging capacity (presented as ABTS value) in the CKE samples was expressed as milligram of Trolox equivalents per gram of dry weight sample (mg Trolox/g dw), and calculated by the calibration equation (y = -0.0007x + 0.6099, R^2 = 0.9994) derived from the calibration curve of the Trolox standard.

The ferric reducing capacity (presented as FRAP value) in the CKE samples was expressed as milligram of Trolox equivalents per gram of dry weight sample (mg Trolox/g dw), and calculated by the calibration equation (y = 0.0052x + 0.1651, R^2 = 0.9992) derived from the calibration curve of the Trolox standard.

The cupric reducing capacity (presented as CUPRAC value) in the CKE samples was expressed as milligram of Trolox equivalents per gram of dry weight sample (mg Trolox/g dw), and calculated by the calibration equation (y = 0.0027x + 0.0922, R^2 = 0.9973) derived from the calibration curve of the Trolox standard.

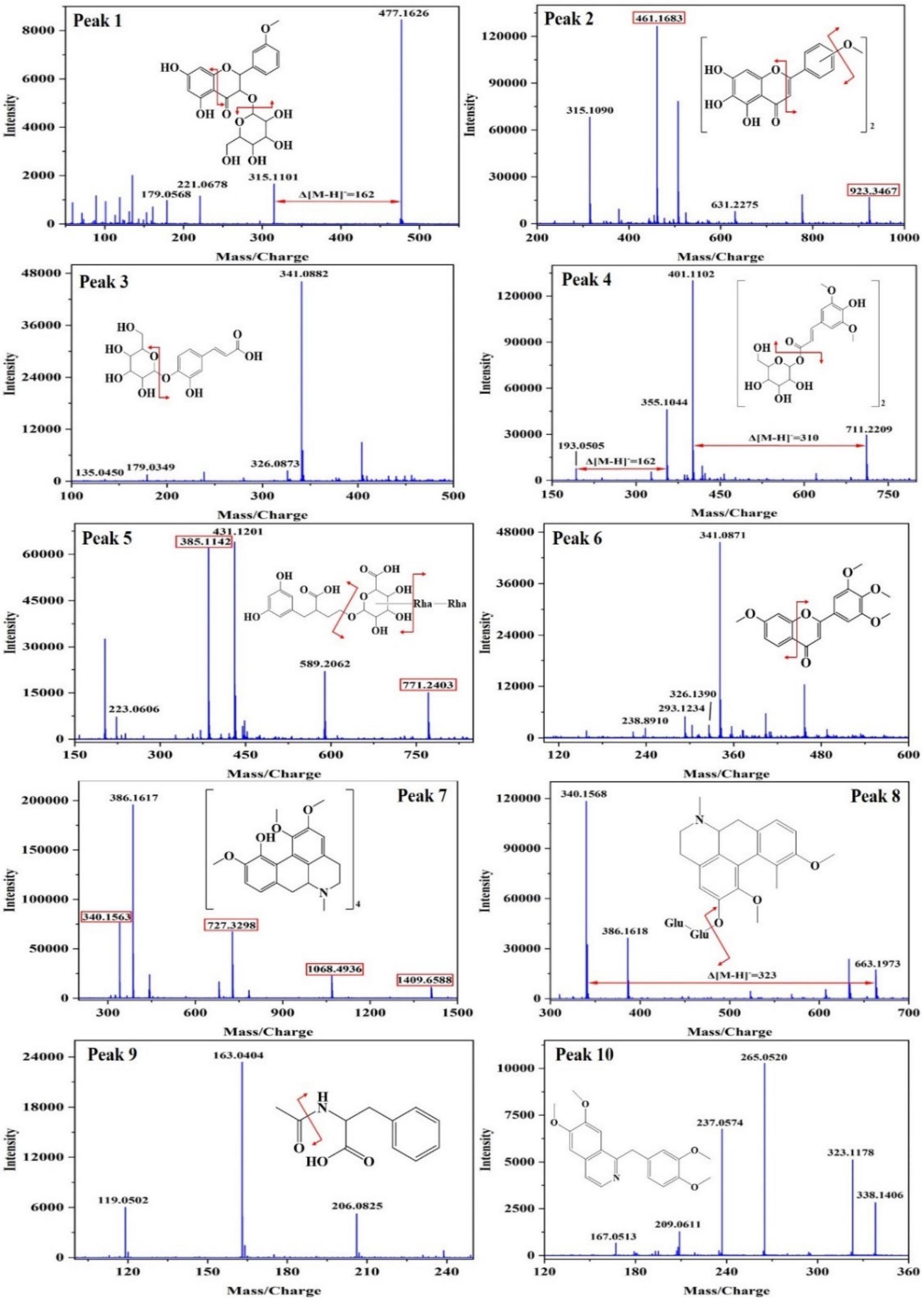

Figure S1. MS^2 spectra and possible fragmentation patterns of tentatively identified compounds in CKE. Glu, glucoside; Rha, rhamnoside. The red boxes indicate potential information about multimers.

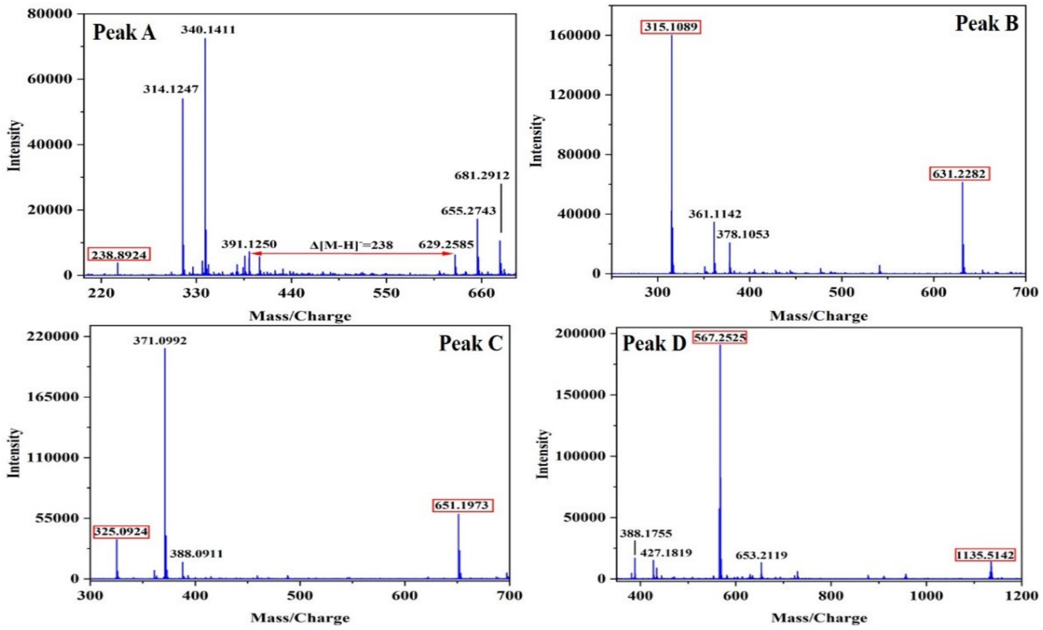

Figure S2. MS^2 spectra of four potential dimers in CKE. The red boxes indicate potential information about dimers.

Table S1. Change of color and browning index in emulsions added with different CKE samples.

Sample	Day	L*	a*	b*	BI
NC	2	92.97	-0.77	-0.97	178.93
	4	92.00	-2.40	1.97	180.75
	7	95.24	0.01	0.38	180.93
	10	94.33	-1.59	1.85	181.24
	13	95.96	-1.98	2.36	181.46
	16	95.86	0.18	0.81	181.50
	19	95.96	0.21	1.61	182.34
	22	97.14	0.23	1.74	182.47
	25	94.50	-0.78	2.52	182.57
	28	96.89	0.11	2.29	182.95
	31	95.65	-1.80	4.88	184.26
EC	2	86.61	4.02	12.11	198.68
	4	84.80	5.03	11.46	199.09
	7	84.61	2.76	13.09	199.38
	10	85.05	7.05	10.63	199.65
	13	92.57	0.00	16.79	200.07
	16	90.34	2.03	15.79	200.98
	19	84.27	6.41	13.73	203.55
	22	85.47	7.57	13.28	203.57
	25	85.20	7.36	13.66	203.99
	28	84.33	7.41	13.88	204.61
	31	86.06	8.23	14.25	205.29
SE	2	90.45	3.44	6.52	190.60
	4	90.16	3.98	6.56	191.12
	7	90.22	3.57	7.67	192.10
	10	90.02	3.97	7.47	192.21
	13	89.82	2.36	8.57	192.25
	16	89.95	4.97	6.98	192.43
	19	89.93	4.19	8.26	193.34
	22	89.14	5.93	7.09	193.45
	25	90.18	4.47	8.33	193.62
	28	89.79	4.71	9.02	194.71
	31	89.37	5.49	11.21	198.14
CP	2	88.88	1.75	4.65	187.20
	4	89.55	2.65	7.46	191.19
	7	88.98	5.18	5.83	191.36
	10	89.75	3.38	7.28	191.54
	13	90.18	4.59	6.94	192.05
	16	90.67	3.88	7.77	192.40
	19	89.86	4.07	7.91	192.84

	22	89.93	4.54	8.06	193.38
	25	89.64	5.13	7.93	193.75
	28	90.05	4.12	8.77	193.89
	31	89.62	4.70	9.15	194.89
AE	2	88.23	1.93	2.90	185.37
	4	91.27	3.23	5.71	189.41
	7	88.83	1.09	7.27	189.76
	10	90.78	3.30	6.87	190.86
	13	87.44	1.23	8.12	191.08
	16	89.46	3.71	7.37	191.95
	19	89.57	3.81	7.36	192.00
	22	90.30	3.62	7.75	192.22
	25	89.60	3.93	7.79	192.61
	28	90.74	3.80	8.06	192.67
	31	90.72	4.63	7.78	193.01
SCFE	2	90.19	2.74	6.79	190.39
	4	90.94	2.83	6.98	190.60
	7	90.46	3.99	6.23	190.70
	10	91.05	2.94	7.47	191.25
	13	91.10	3.84	7.10	191.52
	16	90.75	3.60	7.56	191.92
	19	90.87	4.35	7.39	192.30
	22	90.46	3.50	8.52	193.02
	25	90.92	4.05	9.25	194.27
	28	89.75	4.83	9.61	195.54
	31	90.71	5.06	10.81	197.03

NC, negative control group; EC, catechin group; SE, solvent extraction group; CP, cold pressing extraction group; AE, aqueous extraction group; SCFE, sub-critical fluid extraction group; L*, lightness; a*, degree of redness to greenness; b*, degree of yellowness to blueness; BI, browning index.
